# Influence of Fibrosis Amount and Patterns on Ventricular Arrhythmogenesis and Pumping Efficacy: Computational Study

**DOI:** 10.3389/fphys.2021.644473

**Published:** 2021-06-03

**Authors:** Aulia Khamas Heikhmakhtiar, Abrha Abebe Tekle, Ki Moo Lim

**Affiliations:** ^1^School of Computing, Telkom University, Bandung, Indonesia; ^2^Research Center of Human Centric Engineering, Telkom University, Bandung, Indonesia; ^3^Department of IT convergence Engineering, Kumoh National Institute of Technology, Gumi, South Korea

**Keywords:** fibrosis, arrhythmogenesis, phase singularity, fibrosis entropy, stroke volume

## Abstract

Myocardial fibrosis is an integral component of most forms of heart failure. Clinical and computational studies have reported that spatial fibrosis pattern and fibrosis amount play a significant role in ventricular arrhythmogenicity. This study investigated the effect of the spatial distribution of fibrosis and fibrosis amount on the electrophysiology and mechanical performance of the human ventricles. Seventy-five fibrosis distributions comprising diffuse, patchy, and compact fibrosis types that contain 10–50% fibrosis amount were generated. The spatial fibrosis distribution was quantified using the fibrosis entropy (FE) metric. Electrical simulations under reentry conditions induced using the S1–S2 protocol were conducted to investigate the fibrosis arrhythmogenicity. We also performed mechanical simulations to examine the influence of the fibrosis amount and the spatial distribution of fibrosis on the pumping efficacy of the LV. We observed that the mean FE of the compact type is the largest among the three types. The electrical simulation results revealed that the ventricular arrhythmogenicity of diffuse fibrosis depends on the fibrosis amount and marginally on the spatial distribution of fibrosis. Meanwhile, the ventricular arrhythmogenicity of the compact and patchy fibrosis pattern is more reliant on the spatial distribution of fibrosis than on the fibrosis amount. The average number of phase singularities (PSs) in the compact fibrosis pattern was the highest among the three patterns of fibrosis. The diffuse type of fibrosis has the lowest average number of PSs than that in the patchy and compact fibrosis. The reduction in the stroke volume (SV) showed high influence from the electrical instabilities induced by the fibrosis amount and pattern. The compact fibrosis exhibited the lowest SV among the three patterns except in the 40% fibrosis amount. In conclusion, the fibrosis pattern is as crucial as the fibrosis amount for sustaining and aggravating ventricular arrhythmogenesis.

## Introduction

A cardiac extracellular matrix (ECM) constitutes up to 6% of the myocardium wall. The ECM consists of non-excitable cells called fibroblasts. Fibroblasts produce components that constitute the ECM, e.g., collagen (Rossi, 2001). The ECM provides strength and support to myocytes. In addition, it maintains the myocardial structure and provides a three-dimensional (3D) scaffold (Rossi, 2001; Jugdutt, 2003). Thus, it plays a significant role in determining the mechanical properties of the heart and electrical impulse propagation.

Owing to aging or heart diseases, the amount of the ECM in the myocardium increases substantially and instigates cardiac tissue remodeling. This is because of the proliferation of activated fibroblasts followed by excessive deposition of collagen in the ECM, which is referred to as fibrosis (de Bakker et al., 2005; de Bakker and van Rijen, 2006). The percentage of connective tissue in the heart decreases by up to 40% because of fibrosis (Kawara et al., 2001). Fibrosis occurs mostly to produce a replacement for dead myocytes to preserve the myocardial structure after myocardial infarction. Hence, fibrosis affects the injured areas as well as the myocytes in the vicinity. Other conditions that cause replacement with fibrosis are hypertrophic cardiomyopathy, sarcoidosis, myocarditis, chronic renal insufficiency, and toxic cardiomyopathies (Burt et al., 2014). Fibrosis may occur as a reaction to different cardiomyopathies as those associated with hypertension and diabetes.

Studies have demonstrated that atrial and/or ventricular arrhythmogenesis was increased linearly with an increase in fibrotic tissue (Strain et al., 1983; John et al., 2004; Assomull et al., 2006; Everett and Olgin, 2007; Nakai et al., 2007). Since the fibrotic tissue is not excitable, it is considered that the conduction delay caused by fibrosis plays a significant role in arrhythmogenesis. Conduction delay occurs in areas with an increased amount of fibrosis so the healthy myocytes are isolated from each other by the collagen. Thus, the electrical propagation is compelled to adopt a discontinuous and zigzag pattern through the tissue, from one myocyte strand to another (Gardner et al., 1985; De Bakker et al., 1993). Besides, fibrosis causes unidirectional conduction block owing to the source-sink mismatch between the myocyte strands and neighboring normally coupled tissue (Rushton, 1937; Fozzard and Schoenberg, 1972). The effect of fibrosis in promoting conduction delay and unidirectional blocking generates an environment suitable for reentry initiation. The spatial distribution of fibrosis also exacerbates arrhythmogenicity (Kawara et al., 2001; Tanaka et al., 2007). In general, fibrosis may occur in different distinct forms, namely, diffuse, compact, interstitial, and patchy (De Jong et al., 2011). These forms exhibit different arrhythmogenesis profiles depending on how they affect conduction delay and unidirectional blocking.

Several computational studies have been conducted to better understand the mechanisms of fibrosis arrhythmogenicity. Pertsov (1997) demonstrated that the texture of the fibrotic tissue may cause anisotropic propagation and defragmentation of the scroll wave and alter the rotation of scroll waves. Similarly, Turner et al. (2005) employed a human ventricular tissue model and demonstrated that fibrotic tissue with a string-like form (patchy) causes defragmentation of the electrogram. By using different ion models, other studies have also revealed that diffuse fibrosis increases the vulnerability of tissue to reentry (Panfilov, 2002; Ten Tusscher and Panfilov, 2003; Kuijpers et al., 2005; ten Tusscher and Panfilov, 2005; Spach et al., 2007). Kazbanov et al. (2016) studied the effect of the fibrosis amount and the degree of fibrosis heterogeneity. They observed that the onset of arrhythmias was more likely to increase when the size of fibrosis and the degree of fibrosis heterogeneity were larger.

Although previous studies revealed that fibrosis tissue could result in increased vulnerability to reentry, none of them investigated or quantified the effect of different fibrosis types and fibrosis amount on the perpetuation of arrhythmia and on the cardiac mechanical performance, which is the main function of the heart.

In this study, we investigated the influence of the fibrosis amount and pattern on both arrhythmia sustainability and the pumping performance of ventricles using an electromechanical model. To achieve this, we modeled three categories of fibrosis distribution, which are diffuse, patchy, and compact. In each category, we varied the fibrosis amount from 10 up to 50% with an interval of 10% increment. In each level of the fibrosis amount (10 to 50%), we simulated five unique fibrosis distributions. Hence, we performed 75 individual simulations in total. We quantified the fibrosis entropy (FE) and analyzed the relation of FE with arrhythmia sustainability. We performed electrical simulations under reentry conditions to examine how the various fibrosis distributions and fibrosis densities contribute to the sustainability of arrhythmia. Furthermore, we investigated how electrical instabilities induced by fibrosis affect the mechanical performance, the stroke volume (SV) in particular.

## Materials and Methods

### Model Description

**FIGURE 1:**
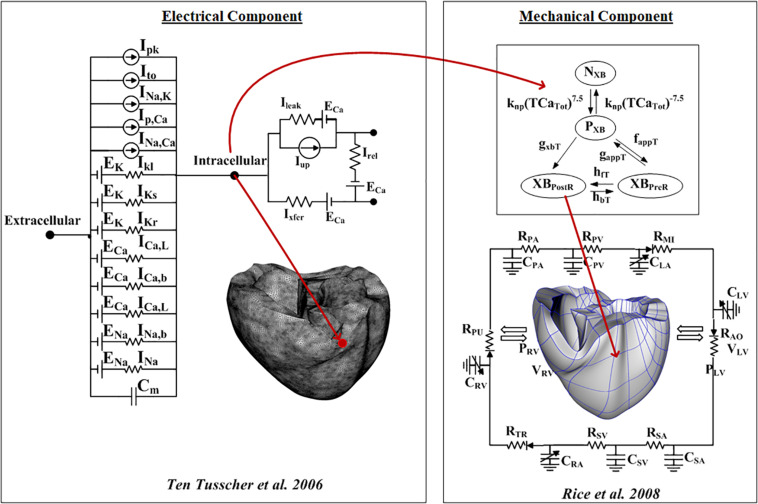
Schematic of the excitation–contraction coupling model of a human ventricular cell. The left side of the diagram is an electrical model that depicts a human ventricular cell model that simulates the ion exchange process through the cell membrane. The electrical model is the Ten Tusscher human ventricular cell model. The mechanical component on the right is the myofilament models developed by Rice et al. (2008).

To investigate the arrhythmogenesis of fibrosis, we employed a ventricular excitation–contraction (E–C) coupling model that comprises the electrophysiological and mechanical properties following the study of Gurev et al. (2010). The electrophysiological conduction model mimics the exchange of ions through the plasma membrane of myocardial cells (Figure 1). The ion model of Ten Tusscher et al. was used to simulate the ventricular cardiomyocyte behavior (Ten Tusscher and Panfilov, 2006). The action potential propagation in myocardial tissue was imparted using the following partial differential equation:

(1) $$\nabla^{.}\tilde{\sigma }\,\nabla V_{m}=\beta \,I_{m}$$

(2) $$I_{m}=C_{m}\,\frac{\partial V_{m}}{\partial t}+\,I_{\text{ion}}\,\left(V_{m,}\,v\right)-I_{\text{trans}}$$

$\tilde{\sigma }$ is the intracellular conductivity tensor, β is the surface–volume ratio of cardiac cells, *I*_*trans*_ is the current density of the transmembrane stimulus, *C*_*m *_ is the membrane capacitance per unit area, *V*_*m*_ is the membrane potential, and *I*_*ion*_ is the current density of the ionic current, which depends on the transmembrane potential and other state variables represented by *v*. *I*_*ion*_ is the sum of all transmembrane ionic currents given by the following equation:

(3) $$I_{i\,o\,n}=I_{N\,a}+I_{K\,1}+I_{t\,o}+I_{K\,r}+I_{K\,s}+I_{C\,a\,L}+I_{N\,a\,C\,a}+I_{N\,a\,K}+I_{p\,C\,a}+I_{p\,K}+I_{b\,C\,a}+I_{b\,N\,a}$$

*I*_*Na*_ is the fast Na^+^ current, I_*K1*_ is the inward rectifier K^+^ current, *I*_*to*_ is the transient outward current, *I*_*Kr*_ is the rapid delayed rectifier current, *I*_*Ks*_ is the slow delayed rectifier current, *I*_*CaL*_ is the L-type Ca^2+^ current, *I*_*NaCa*_ is the Na^+^/Ca^2+^ exchanger current, *I*_*NaK*_ is the Na^+^/K^+^ pump current, and *I*_*pCa*_ and *I*_*pK*_ are the plateau currents of Ca^2+^ and K^+^, respectively. *I*_*bCa*_ and *I*_*bK*_ are the background currents of Ca^2+^ and K^+^, respectively. The equations for these ionic currents are specified in the membrane current; please refer to Ten Tusscher and Panfilov (2006) for details on each equation. Finite tetrahedral linear elements were generated by using the HyperMesh software (214,319 nodes and 1,061,379 elements). For the electrical heart model, we used the isotropic condition with the fiber orientation shape following Gurev et al. (2010).

To simulate the fibrotic condition in the myocytes, the property of the electrical model was modified in the fibrotic nodes. The action potential fibrosis model was adapted from the model of Ten Tusscher and Panfilov (2006) by scaling 50% reduction in *I*_*K1*_; 50% reduction in *I*_*CaL*_; and 40% reduction in *I*_*Na*_ (Corradi et al., 2008;Nattel et al., 2008). Furthermore, the conductivity was reduced by 30% in the fibrotic regions to simulate the conduction delay caused by fibrosis (Li et al., 1999; Burstein et al., 2009). In this study, we investigated the influence of conduction velocity (CV) in the fibrosis models to expand the analysis. To regulate the CV, we scaled the diffusion coefficient, which represents conductivity in the electrical propagation.

Excitation–contraction coupling was implemented by applying the calcium transient data extracted from the electrophysiological simulation to the mechanical simulations to induce contraction of the myofilaments and generate tension through the Ca^2+^-induced Ca^2+^-release (CICR) current. The mechanical cross-bridge cycling model provided by Rice et al. (2008) was applied to simulate the mechanical cardiac response. The calcium dynamics to generate the calcium transients can be represented using the following equations:

(4) $$\frac{{\mathrm{dCa}}_{\text{itotal}}}{\mathrm{dt}}=-\frac{I_{\mathrm{Ca},L}+I_{b,\mathrm{Ca}}+I_{p,\mathrm{Ca}}-2\,I_{\mathrm{Na},\mathrm{Ca}}}{2\,V_{C}\,F}+I_{\text{leak}}-I_{\text{up}}+I_{\text{rel}}$$

(5) $$\frac{{\mathrm{dCa}}_{\text{srtotal}}}{\mathrm{dt}}=\frac{V_{c}}{V_{\mathrm{SR}}}\,\left(-I_{\text{leak}}+I_{\text{up}}-I_{\text{rel}}\right)$$

where *Ca*_itotal_ is the total amount of calcium in the cytoplasm, and *Ca*_srtotal_ is the total amount of calcium in the sarcoplasmic reticulum (SR). *I*_rel_ is the calcium current released from the junctional SR (JSR), and *I*_leak_ is the leakage calcium current of the JSR. *I*_*up*_ is the absorbed calcium current in the network SR (NSR), and *I*_xfer_ is the diffusible calcium current between the dyadic subspace and bulk cytoplasm.

The mathematical description of the mechanical contraction of cardiac tissue is based on continuum mechanics (Guccione et al., 1995; Usyk et al., 2002), with the myocardium assumed to be hyperelastic, nearly incompressible material, the passive mechanical properties of which were defined by an exponential strain function (Usyk et al., 2002).

(6) $$W=\frac{C}{2}\,\left(e^{Q}-1\right)$$

(7) $$Q=b_{1}\,E_{\mathrm{ff}}^{2}+b_{2}\,\left(E_{\mathrm{rr}}^{2}+E_{\mathrm{cc}}^{2}+2\,E_{\mathrm{rc}}^{2}\right)+2\,b_{3}\,\left(E_{f\,r}^{2}+E_{f\,c}^{2}\right)$$

where *W* is a strain energy function, and the Langian Green’s strains E_ij_ are referred to the local fiber coordinate system. C is 2kPa, b_f_ is 8, b_t_ is 2, and b_fs_ is 4. The laminar sheet and fiber orientation information determined the orthotropic electrical conductivity and passive mechanical properties of the myocardium. We used a finite element mesh consisting of 462 nodes and 230 Hermite elements. The cross-bridge dynamics model (Rice et al., 2008) represented the generation of active tension at the level of a single myocyte.

To simulate hemodynamic responses, which are the interactions between the blood and the ventricles, the finite element electromechanical model of the human ventricle was coupled with a circulatory model using the coupling method of Gurev et al. (2010). A schematic diagram of the integrated model is illustrated in Figure 1.

### Fibrosis Distribution

In this study, we investigated the three fibrosis patterns such as diffuse, patchy, and compact. The diffuse pattern representing the fibrotic cell that was randomly spread out in the ventricular tissue. The fibrotic cells were applied randomly in the 3D ventricular model for the diffuse type depending on its level (10 to 50%). The patchy type of the distribution was modeled to exhibit a striped shape in the ventricular medium. The areas of patchy fibrosis were determined manually such that the fibrosis regions isolate cardiomyocytes over extended distances. For the compact type, the region of fibrosis was generated by forming various groups of fibrosis regions in different locations of the ventricular mesh. This fibrotic region was generated by a program that assigns a group of nodes that are linked together. Furthermore, we simulated five different fibrotic distributions for each fibrotic pattern and amount. Hence, in total, we simulated 75 ventricular models.

### Quantification of Spatial Distribution of Fibrosis

To better understand the spatial fibrosis distribution in each ventricular model, we calculated the local FE of each element. It was computed by considering the characteristics of the local tissue element and neighboring elements. The local FE of the ith element was calculated to indicate the level of chaos within the element and its neighboring elements. It was expressed using the Shannon entropy eq. (10) (Zahid et al., 2016).

(8) $$\mathrm{FE}=\sum_{i=1}^{N}\frac{-P_{i}\,\ln\,\left(P_{i}\right)}{N}$$

*N* denotes the i^th^ element and all its neighboring elements. *FE* is the total number of fibrotic elements that are neighbors of the ith element. *P*_i_ is the ratio of the number of elements that are different from the ith element to the total number of neighboring elements. The computation of mean FE for each fibrosis type involved the following procedures. First, the local FE of all the nodes in a ventricle was computed according to the spatial fibrosis distribution in each ventricular model. Second, the mean FE (MFE) of all the nodes was calculated. Because there are five different distributions in each fibrosis type that have equal fibrosis amounts, the average FE of these five distributions was then calculated.

### Simulation Protocols

To assess the effect of the fibrosis amount and spatial distribution, a series of 3D electromechanical simulations were conducted under the reentry condition. In the electrical simulation, reentry was initiated using the S1–S2 protocol. First, three S1 electrical stimuli were applied at the apex of the ventricle with a 600-ms cycle length. This produced a planar wavefront that propagated toward the base of the ventricle. When the wavefront of the last S1 stimulus reached half of the ventricle, the left side of the ventricle was artificially reset to the resting state of the membrane potential. This caused the wavefront to propagate toward the artificially reset region, producing a spiral wave.

To investigate the range of conduction velocity (CV) that sustains the reentrant wave, we simulated all the 75 ventricular models within the CVs of 53 and 100 cm/s with the interval of 3 cm/s. The results were then put into the binary condition whether the reentry was sustained for 10 s or not. After we obtained the maximum CV that sustained the reentrant wave, we quantified the electrical instability caused by fibrosis. To achieve this, we calculated the phase singularities (PSs) from the results of the electrical simulations using the Iyer and Gray method (Iyer and Gray, 2001). Furthermore, we investigated the ventricular pumping performance based on the electrical simulation with the maximum range of the CV that sustained the reentrant wave.

To simulate the electromechanical coupling model, we extracted the calcium transient data from its respective 3D electrophysiological fibrosis condition and used the calcium data as input to the mechanical simulator following Gurev et al. (2010). Lastly, we compared the SV of all cases.

## Results

### Fibrosis Distributions

**FIGURE 2:**
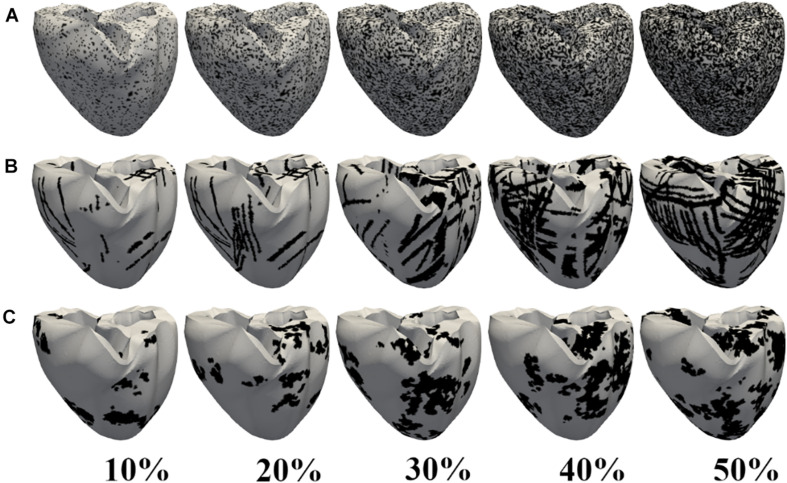
Fibrosis patterns generated. Diffuse **(A)**, patchy **(B)**, and compact **(C)** fibrosis patterns. The black regions indicate areas of fibrosis.

Figure 2 shows the representation of the diffuse (2A), patchy (2B), and compact (2C) fibrosis patterns from 10 to 50% fibrosis amount. In the diffuse pattern, small fibrotic tissue was interspersed randomly among myocytes in all parts of the ventricle. In the patchy pattern, these fibrosis regions do not entirely consist of fibrotic nodes. Some nonfibrotic nodes were also embedded. Thereby, normal myocytes were loosely interconnected to each other. In the compact fibrosis pattern, fibrosis tissue exists as a large dense area of fibrosis. These dense fibrotic regions mimic large scars in the myocardium caused by myocardial infarction or other chronic diseases. The large scars are devoid of cardiac myocytes.

### Quantification of Fibrosis Spatial Distributions

**FIGURE 3:**
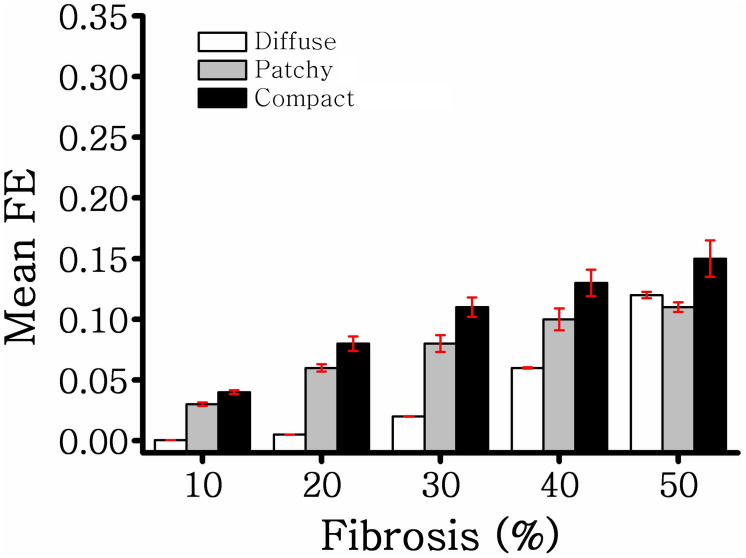
Mean fibrosis entropy of diffuse, compact, and patchy fibrosis.

Figure 3 illustrates the MFE of five different samples in diffuse, compact, and patchy fibrosis patterns with fibrosis amount of 10–50%. In general, the MFE was increased in all fibrosis patterns along with the fibrosis densities. The MFE of the compact patterns was the highest among the three patterns. This shows that the fibrosis in the compact pattern has the most fibrosis intensity. When the fibrosis amount was between 10 and 40%, the patchy fibrosis ventricles exhibited higher MFE than the diffuse ones. However, at 50% of the fibrosis amount, the diffuse patterns exhibited larger MFE than the patchy ones. The reason was that under the 50% fibrosis amount, the diffuse pattern has a high amount of fibrosis spread out in the neighboring nodes, and the region of the normal tissue was small. While in the patchy pattern, the region of the normal tissue still remained larger compared to that in the diffuse pattern (Figures 2A,B).

### Arrhythmia Sustainability

**FIGURE 4:**
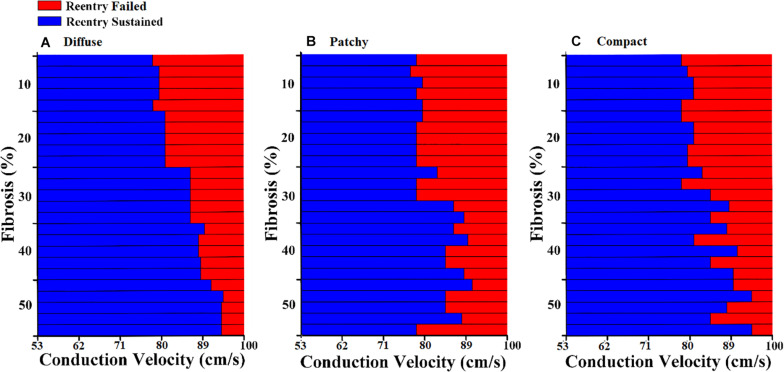
Conduction delay owing to fibrosis distribution and fibrosis amount of **(A)** diffuse fibrosis, **(B)** patchy fibrosis, and **(C)** compact fibrosis.

The diffuse fibrosis patterns revealed that the CV required to sustain the induced reentrant wave was increased along with the fibrosis amount, from 10 to 50% (Figure 4A). However, in the 10 to 30% of the fibrosis amount, the CV required to sustain the reentry wave was approximately the same, 79 cm/s. In the 40 and 50% of the fibrosis amount, the variation in the CV required to maintain the reentry was marginally increased.

In the patchy pattern, the CV required to sustain the reentrant wave increased inconsistently with the fibrosis amount (Figure 4B). The required CV that sustained the reentrant wave was beginning to increase in the 30% fibrosis amount. Arrhythmogenesis in the patchy pattern was more reliant on the spatial distribution of fibrosis than on the fibrosis amount. Furthermore, simulations under an equal fibrosis amount with different distributions displayed different values of the required CV for reentry to sustain. In the compact fibrosis pattern, the CV required to sustain a reentry wave was increased along with the fibrosis amount.

### Electrical Instability Owing to Fibrosis

**FIGURE 5:**
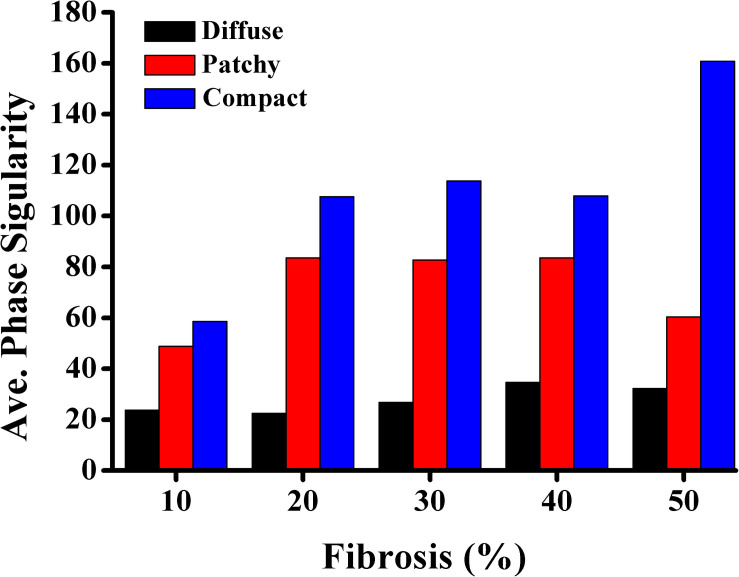
Average number of phase singularities.

PS represents the core of a spiral wave or a rotor in ventricular fibrillation. We calculated the number of PSs to characterize the electrical instability induced by fibrosis. The average number of PSs during 10 s of simulation time was employed for analysis. Figure 5 presents the average number of PSs caused by diffuse, patchy, and compact fibrosis types. For all the fibrosis amounts considered in this study, the average number of PSs in the compact fibrosis was the highest among the three patterns, with the diffuse pattern being the lowest.

**FIGURE 6:**
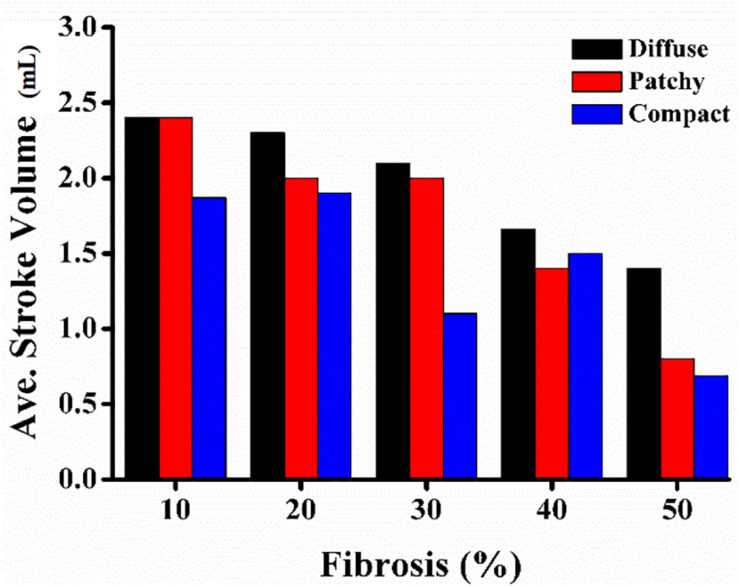
Average left-ventricle stroke volume.

Figure 6 shows the correlation between MFE and PS under diffuse, patchy, and compact fibrosis patterns. The correlation between the two parameters of that in patchy patterns showed 0.3864, while the correlations between MFE and PS under the diffuse and compact patterns were 0.8228 and 0.9022, respectively.

### Mechanical Results

**FIGURE 7:**
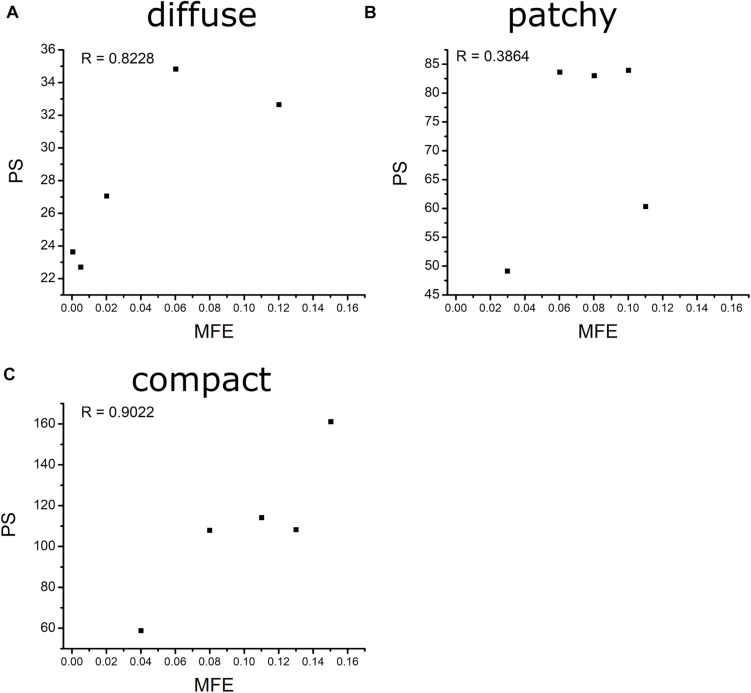
Correlation between MFE and MPS of diffuse **(A)**, patchy **(B)**, and compact **(C)** fibrosis patterns.

Figure 7 illustrates the average SV under diffuse, patchy, and compact fibrosis patterns. Overall, there was a significant reduction in the SV as the fibrosis amount in the ventricular model increased. The SV under the diffuse pattern was the highest among the three patterns. The SV under compact fibrosis was the lowest among the three types of fibrosis, except when the fibrosis amount was 40%. The reduction in the SV was highly correlated to the electrical instabilities induced by the fibrosis amount and the spatial distribution of fibrosis. The higher impact of compact fibrosis on electrical instability resulted in a lower SV, whereas the lower electrical instability induced by diffuse fibrosis resulted in a higher SV. Patchy fibrosis marginally affected the electrical instability and also marginally reduced the mechanical performance of the left ventricle (LV).

## Discussion

In this study, we investigated the effect of the fibrosis amount and the spatial fibrosis distribution on electrical phenomena and the mechanical performance of ventricles under reentry conditions by employing a human ventricular cardiomyocyte model. To our knowledge, this is the first study that investigated the effect of the fibrosis amount and fibrosis patterns on the pumping capacity of the heart. The key observations of this study are as follows:

1. The CV required to sustain the reentry was increased consistently with an increase in the fibrosis amount under the diffuse pattern in the ventricular model. However, this correlation between the CV and the fibrosis amount was not consistent in the patchy and compact fibrosis types.
2. The average number of PSs in the compact fibrosis was the highest among the three fibrosis patterns. The reason was the region of normal myocardium cells was sufficient for PS to be generated since the fibrosis cells were concentrated in some specific region. While in diffuse and patchy patterns, the fibrosis cells were more evenly spread out.
3. The average SV under compact fibrosis was the lowest, which associated with the high number of MFE.
4. There was a strong correlation between MFE and PS under the compact and diffuse fibrosis patterns but not in the patchy pattern.

Because fibrosis architecture varies across patients (Marrouche et al., 2014), we generated 75 different fibrosis distributions comprising diffuse, compact, and patchy fibrosis patterns based on Nguyen et al. (2014). In diffuse fibrosis, short collagen septa are interspersed among myocytes. Patchy fibrosis patterns were represented as collagen fiber embedded among myocytes over a long distance. Compact fibrosis patterns were simulated as a large accumulation of fibrotic tissue devoid of healthy myocytes that resembles a large scar caused by myocardial infarction and other diseases. The maximum amount of fibrosis in a myocardial wall used in our study was 50%. This was based on the result of a previous study, which stated that the fibrosis amount in a chronically diseased heart may increase by up to 43% (Kawara et al., 2001). We developed a 3D map of the local FE of each element in the ventricular model to visualize the level of disorganization of the fibrosis spatial patterns. Our results demonstrated that among the three fibrosis patterns employed in this study, the compact fibrosis pattern exhibited the highest MFE. When the fibrosis amount was 40% or less, the compact pattern exhibited the second highest MFE. However, at 50% fibrosis, diffuse fibrosis tends to exhibit a higher value of MFE than patchy fibrosis. High MFE is associated with ventricular regions featuring a high degree of intermingling between fibrotic and nonfibrotic tissue. Hence, the fibrosis distribution in the compact fibrosis is highly vulnerable, whereas diffuse fibrosis is less.

The arrhythmogenic properties of the fibrosis patterns were analyzed by electrophysiological simulations under reentry induced by the S1–S2 protocol. An S2 stimulation was preceded by three S1 stimulations from the apex of the ventricle. The 3D electrophysiological simulation responses under the reentry condition revealed that the CV required to sustain the reentrant wave increased consistently as the diffuse fibrosis amount increased from 10 to 50% (Figure 4). This correlation between arrhythmogenesis and diffuse fibrosis amount is a result of small fibrosis tissue interspersed among cardiomyocytes throughout the ventricular model. It is highly unlikely that fibrosis is prevalent in a region, and less prevalent or absent in other regions. Therefore, ventricular models that had equal amounts of fibrosis exhibited similar fibrosis textures (Figure 2). Figure 3 also shows that diffuse fibrosis exhibited the least MFE among the fibrosis patterns. Consequently, simulations with equal amounts of diffuse fibrosis albeit different distributions exhibited almost similar arrhythmogenic profiles. This observation agrees with the report of Kazbanov et al. (2016) that the arrhythmogenesis of diffuse fibrosis depends on both the amount of fibrosis and the spatial distribution of fibrosis.

In contrast, a strong correlation between the fibrotic spatial distribution and the arrhythmogenic effect was apparent in electrical simulations under patchy and compact fibrosis forms. In these forms, the correlation between the fibrosis amount and ventricular arrhythmogenesis is nonlinear. The higher MFE of patchy and compact fibrosis patterns caused simulations with equal fibrosis amounts to exhibit different arrhythmogenesis. In certain simulations, a marginal amount of patchy fibrosis tended to exhibit higher arrhythmogenic potential than a large amount of patchy fibrosis. This is owing to the non-uniform distribution of fibrosis in the ventricular models, notwithstanding the identical overall fibrosis amount. This effect was more aggravated in compact fibrosis patterns. During reentry, the activation wave anchors and rotates around PS points. We detected PS during the electrophysiological simulation under fibrosis. We demonstrated that the average PS was the highest in the electrophysiological simulations under compact fibrosis than those under patchy and diffuse fibrosis. The lowest number of PSs was detected in the electrophysiological simulations under diffuse fibrosis. We also examined the effect of electrical instability induced by fibrosis, on the mechanical performance of the LV. Our results demonstrated that the ventricular models with compact fibrosis distributions yielded the lowest average SV, whereas those with diffuse fibrosis yielded the highest average SV among the three fibrosis types. The electrical perturbation predisposed by fibrosis significantly impacted the reduction in the pumping efficacy of the LV.

Fibrosis cells in the myocardium disturb the sequence of the electrical activity by conduction block. Hence, a high number of fibrosis cells in the myocardium increase the risk of arrhythmia. Furthermore, the distribution pattern of the fibrosis contributes to the behavior of the arrhythmia as well. In this study, we analyzed the effect of the fibrosis amount and the three fibrosis patterns on the sustainability of the arrhythmia in a range of CV. The three patterns were quantified by measuring the mean FE and the PS. The MFE indicated the amount of fibrosis in one area, while the PS indicated the number of scroll waves that occurred in the ventricles. The PS in the compact pattern showed the highest number of PSs among the three patterns especially at 50% of the fibrosis amount. The reason for this is that in the compact pattern, the fibrosis cells were concentrated in more specific areas, thus the wide range of normal myocardium cells giving sufficient space for PS to occur. While in the other patterns, the fibrosis cells were scattered especially in the diffuse pattern, which inhibits the PS occurrence. As result, the compact pattern showed the poorest heart performance indicated by the highest MFE, PS, while having the lowest SV. However, in the other fibrosis distribution patterns, the heart performance was significantly reduced when the fibrosis amount is more than 30%. Furthermore, our results showed that the MFE and PS have a strong correlation on both compact and diffuse patterns with R equals 0.9022 and 0.8228, respectively, while the correlation between the MFE and PS was low in the patchy pattern.

Our study had several limitations to be addressed. First, we did not validate the integrated model of the electromechanical ventricle used in this study by disregarding the baseline or normal heart condition. Second, in this fibrosis model, we did not consider other parameters such as alternans, ERP, and repolarization heterogeneity. Third, the fibrosis model in this study was based on the fibrosis remodeling of the atrium cell. Fourth, the electrical conductivity throughout the tissue was considered isotropic. Furthermore, we employed one-way coupling by extracting Ca^2+^ information from electrical activation and furnished it to the mechanical stimulation. The feedback from the mechanical stimulation to the electrical stimulation owing to stretch-activated channels was not considered.

## Conclusion

Fibrosis inhibits the electrophysiological properties of ventricles by diminishing the gap junction between myocytes. Abundant fibrosis tissue in the myocardium is associated with the sustainability of the reentry condition. In addition, the spatial distribution of fibrosis also plays a major role in determining arrhythmogenesis. Overall, the likelihood of sustaining reentry in the 3D ventricular model under the reentry condition was higher as the intensity of fibrosis in the ventricular model increased in all the fibrosis patterns considered in this study. Variation in the spatial distribution of fibrosis substantially affected the arrhythmogenesis of patchy and compact fibrosis other than that of the diffuse fibrosis pattern. The electrical instability caused by fibrosis was characterized by an average number of PSs. Hence, the average number of PSs detected in the compact fibrosis pattern was the highest among the three patterns, while the average number of PSs detected under the diffuse fibrosis pattern was the lowest. As a result, the SV under the compact fibrosis was the lowest, while the SV under the diffuse pattern was the highest.

## Data Availability Statement

The original contributions presented in the study are included in the article/supplementary material, further inquiries can be directed to the corresponding author/s.

## Author Contributions

This manuscript is the intellectual product of the entire team. AT contributed toward the performance of the simulation, data analysis, and manuscript drafting. KL contributed toward the development of the research concept, simulation design, and development of the simulation source code. All authors contributed to the article and approved the submitted version.

## Conflict of Interest

The authors declare that the research was conducted in the absence of any commercial or financial relationships that could be construed as a potential conflict of interest.
